# Effect of Switching Antiretroviral Treatment Regimen in Patients With Drug-Resistant HIV-1 Infection: Retrospective Observational Cohort Study

**DOI:** 10.2196/33429

**Published:** 2022-06-24

**Authors:** Yiping Li, Qinjian Wang, Shu Liang, Chuanteng Feng, Hong Yang, Hang Yu, Dan Yuan, Shujuan Yang

**Affiliations:** 1 Center for AIDS/STD Control and Prevention Sichuan Center for Disease Control and Prevention Chengdu China; 2 West China School of Public Health and West China Fourth Hospital Sichuan University Chengdu China; 3 Institute for Disaster Management and Reconstruction Sichuan University Chengdu China; 4 International Institute of Spatial Lifecourse Health Wuhan University Wuhan China

**Keywords:** HIV, antiretroviral therapy, drug resistance, protease inhibitors, parametric g-formula

## Abstract

**Background:**

Evidence on the efficacy of antiretroviral therapy (ART) regimen switches on the mortality of patients with HIV drug resistance (HIVDR) is limited.

**Objective:**

We aim to provide policy guidance for ART regimen selection and evaluate the effectiveness of ART regime switches for people living with HIV and HIV-1 drug resistance.

**Methods:**

This retrospective observational cohort study included 179 people living with HIV and HIV-1 drug resistance from 2011 to 2020. The time that participants switched treatment regimens either to protease inhibitor (PI)–based ART regimens (PIs) or nonnucleoside reverse transcriptase inhibitor (NNRTI)–based ART regimens (NNRTIs) was taken as an observation starting point and followed up every 12 months. The parametric g-formula was used to estimate the 5-year risk of mortality under the situations of (1) natural course, (2) immediate switch to NNRTIs, (3) immediate switch to PIs, and (4) if CD4(+) T cells<200 switched to PIs.

**Results:**

The follow-up time of the 179 patients ranged from 30 to 119 months. The median follow-up time was 90 months. During a follow-up of 15,606 person-months, 27 individuals died in the cohort. The estimated 5-year risk of mortality under natural course, immediate switch to NNRTIs, immediate switch to PIs, and if CD4(+), and switch to PIs if T cells<200 were 11.62% (95% CI 7.82-17.11), 31.88% (95% CI 20.79-44.94), 2.87% (95% CI 0.32-7.07), and 5.30% (95% CI 2.07-10.21), respectively. The risk ratios (RRs) of immediate switch to NNRTIs, immediate switch to PIs, and switch to PIs if CD4(+) T cells<200, compared with natural course mortality rate, were 2.74 (95% CI 2.01-3.47), 0.25 (95% CI: 0.04-0.54), and 0.46 (95% CI 0.22-0.71), respectively. The risk differences were 20.26% (95% CI 10.96-28.61), –8.76% (95% CI –13.34 to –5.09) and –6.32% (95% CI –9.75 to –3.11), respectively.

**Conclusions:**

Our study found that a PI-based ART regimen was beneficial for reducing mortality in people living with HIV and HIV-1 drug resistance. More effort should be given to find HIV-1 drug resistance earlier to ensure a timely adjustment to PI-based ART, thereby maximizing the benefit of early switch treatment for people living with HIV and HIV-1 drug resistance.

## Introduction

The widespread use of antiretroviral therapy (ART) has effectively prolonged the life span of people living with HIV and has reduced the risk of HIV transmission [1]. However, many challenges have emerged with the promotion and use of ART [2,3]. HIV drug resistance (HIVDR) is a critical cause of virological failure in people living with HIV. It compromises the therapeutic effects for individuals and endangers the population [4]. Between 2016 and 2030, a pretreatment HIVDR level of over 10% (mean 15%), 16% of AIDS deaths, 9% of new infections, and 8% of ART program costs in sub-Saharan Africa will be attributable to HIVDR [5]. In limited-income countries, ART failure based on nonnucleoside reverse transcriptase inhibitors (NNRTIs) occurs in 10%-30% of people living with HIV per year [6-8].

A protease inhibitor (PI)–based ART regimen includes 2 nucleoside reverse transcriptase inhibitor (NRTI) drugs (zidovudine/tenofovir and lamivudine, TDF/AZT and 3TC), and 1 of the PI drugs (lopinavir and ritonavir; LPV/r) [9]. Boosted PI options are currently recommended as part of second-line regimens due to their safety and efficacy, as proven by systematic reviews and meta-analyses [10,11]. Patients with HIVDR on NNRTIs should, in principle, switch to PIs as soon as possible, as a delay in switching treatment regimens has led to increased mortality [12-16]. However, it is still common to delay the switch [17,18]. Several observational studies have investigated the estimated effect of the delayed switch to PIs on mortality. Tsegaye et al [15] found that the risk of death was 4.8 times higher for people with HIV who did not switch to PIs than those who did switch. Gsponer et al [16] showed a drastic reduction in mortality for patients who switched to PIs compared to those who did not based on an immunological criterion of failing and the benefit of switching early. Petersen et al [19] estimated that among HIV-infected patients with confirmed virologic failure on NNRTIs, remaining on NNRTIs led to an increase in mortality relative to switching to PIs.

In China, only a few studies have compared the differences in immunological outcomes and drug resistance between PIs and NNRTIs among people living with HIV [20,21], more so for people living with HIV who developed resistance to NNRTIs, as not all of them could immediately switch to PIs and instead switched to other NNRTIs due to limited health resources. However, no studies have compared the mortality difference between switching to other NNRTIs and switching to PIs in China. Since information on the effect of ART regimen switches on the mortality of patients with HIV is limited, research is warranted to accurately judge ART regimen switches and guide the regimen selection for optimal treatment.

To fill these research gaps, we conducted a 9-year retrospective cohort study to compare the impact of switches to other NNRTIs and switches to PIs on mortality in Sichuan, where the largest population of people with HIV resides in China [22]. The parametric g-formula adjusted the time-varying confounders affected by previous treatments [23]. We chose the parametric g-formula since traditional multivariate regression techniques may yield biased treatment effect estimates in our context. In contrast, the parametric g-formula can appropriately adjust for measured time-varying confounders. This research aims to guide policies on ART regimen selection and evaluate the effectiveness of ART regimen switches in people living with HIV and HIV-1 drug resistance.

## Methods

### Study Design and Participants

A retrospective observational cohort study was conducted based on the National Free Antiretroviral Treatment Program database of Sichuan. This confidential, nonpublic database is managed by the Chinese Center for Disease Control and Prevention. Each province, municipality, and autonomous region has access to data within its jurisdiction.

Participants of this study were selected from the database according to the following inclusion criteria: people living with HIV who (1) were under NNRTIs based ART regimens for 12 months in Sichuan during 2011-2014; (2) failed those NNRTI-based ART regimens with viral load ≥1000 copies/ml; (3) were tested for HIV-1 genotype resistance and confirmed to have HIV-1 drug resistance; and (4) received at least 1 test of CD4(+) T cells and viral load during the follow-up period. The exclusion criteria included people living with HIV (1) without immunological outcomes after switching ART regimen during the follow-up period; and (2) who did not have the demographic information (eg, gender, age, and ART) in the baseline database. A total of 2037 people living with HIV tested for HIV-1 genotype resistance, and 197 were confirmed to have HIV-1 drug resistance. Of these 197 people with HIV, 18 were excluded without detecting CD4(+) T cells and viral load during the follow-up period. Finally, 179 people with HIV were included in the final analysis.

In 2011, the first person living with HIV entered the cohort, and the last one entered in 2014. The starting point of observation was defined as when the person with HIV switched treatment regimens, and each person living with HIV was followed up from the entry date to the date of death or the end of this study (December 2020). A total of 179 participants met the inclusion criteria and were included in this study. They were followed up approximately every 12 months for documentation of their CD4(+) T cell count, viral load, and drug resistance. We estimated and compared the mortality risks of 3 simulated ART regimen switch scenarios with the real-world scenario (natural course), including (1) immediate switch to NNRTIs, (2) immediate switch to PIs, and (3) switch to PIs if CD4(+) T cells<200

### Ethics Approval

The Ethical Committee of Sichuan Center for Disease Control and Prevention approved this study (No. SCCDCIRB2021-025). This study was conducted in accordance with the Declaration of Helsinki.

### Data Collection

The NNRTI and PI regimens were carried out following the approved guidelines [24]. NNRTI regimens consisted of tenofovir/zidovudine (TDF/AZT) + lamivudine (3TC) + efavirenz/ nevirapine (EFV/NVP). PI regimens included tenofovir/zidovudine (TDF/AZT) + lamivudine (3TC) + lopinavir and ritonavir (LPV/r).

The CD4(+) T cell count and viral load were collected at the starting point every 12 months of follow-up to evaluate the immunological reconstruction effect. ART regimens and survival status were also included in the follow-up. To deal with missing CD4(+) T cell count during the followed-up period, we used the expectation-maximization-bootstrap algorithm for multiple imputations [25]. The imputation model included all baseline and follow-up variables (including lagged and lead versions). The algorithm accounted for the nonlinear and longitudinal structure of the data.

### Covariates

All baseline characteristics were taken as covariates—demographic and HIV-related characteristics and immunological outcomes. Demographic characteristics included age, gender, and education level. HIV-related characteristics included transmission patterns, history of sexually transmitted diseases, and history of tuberculosis treatment.

### Laboratory Tests

All participants provided blood specimens to measure CD4(+) T cell count at the starting point and during follow-up after switching treatment regimens, measured using flow cytometry (FACSC Calibur, BD). Real-time molecular beacon detection was applied to detect the viral load of HIV (NucliSens EasyQ Analyzer). Reverse transcription-polymerase chain reaction (RT-PCR) was used to amplify a 1300-bp fragment of the HIV pol gene for drug resistance mutation analysis and viral subtype determination. Successfully amplified sequences were analyzed for HIVDR using the Stanford University HIV Drug Resistance Database. All experimental protocols followed the manufacturer's instructions. People living with HIV with low or higher drug resistance to one or more drugs were considered as having HIVDR [26-28].

### Statistical Analysis

Adjustment is usually used for confounders in regression models (eg, the Cox proportional hazards model), which is equivalent to estimating the hazard ratios of a specific stratum and then averaging the information-weighted hazard ratios. When some of these confounding factors are also causal intermediates, the effects of exposure are adjusted [29]. However, the first step in g-formula is to obtain the weighted averages of the stratum-specific hazards and then combine the averaged (standardized) hazards into a summary hazard ratio. The potential bias arising from time-varying covariates that can be both confounders and causal intermediates is a drawback of using regression models [30,31], which can be overcome by using the g-formula [32]. This study considers that patients' treatment regimens differed by their CD4(+) T cell count and viral load, which were also influenced by their previous treatment regimens and other baseline covariates. The outcomes (death) were influenced by time-varying treatment regimens and time-varying covariates (eg, CD4(+) T cell count and viral load level), and baseline characteristics (Multimedia Appendix 1). Our estimates had to adjust the time-varying confounders CD4(+) T cell count and viral load level and confounders measured at baseline. Since standard statistical methods cannot appropriately adjust for time-varying confounders affected by previous ART treatment [23], we applied the parametric g-formula to obtain adjusted estimates (eg, mortality risk) for each treatment strategy under the assumptions of conditional exchangeability, positivity, no residual confounding, no measurement error, and no model misspecification [32,33].

Specifically, the procedure of the parametric g-formula had 3 steps. First, we fit separate logistic regression models for the treatment and viral load and linear regression models for CD4(+) T cell count. All regression models included time-varying covariates (treatment, viral load level, and CD4(+) T cell count) and baseline variables (age, gender, education level, marital status, patterns of transmission, history of sexually transmitted diseases, and history of tuberculosis treatment). The assumed relationships between all variables are depicted in Multimedia Appendix 1. Second, a pseudo sample more prominent than the overall sample size, set as 10,000 in this study, was generated by Monte Carlo simulation based on the distribution of the postbaseline outcomes and time-varying covariates separately under each ART regimen switch scenario. Third, a bootstrap sampling method was used to repeat the aforementioned process 500 times to obtain the 95% CIs [34].

The RRs and risk differences (RDs) and their 95% CIs were estimated to compare the mortality risk between the natural course and 3 hypothetical ART switch strategies. To explore the validity of our parametric assumptions, we compared the observed (nonparametric estimates) means of the outcome and time-varying covariates with those predicted by our models (parametric g-formula estimates) (Multimedia Appendix 2). All statistical analyses were performed using R 4.0.3 (R Foundation for Statistical Computing).

### Sensitivity Analyses

A total of 4 sensitivity analyses were performed in this study to ensure the stability of the results. First, we excluded people older than 60 years at baseline since they may have a higher risk of death. Second, CRF01_AE was the primary subtype of people living with HIV in Sichuan; thus, we restricted to the subset of participants with the CRF01_AE subtype to estimate the mortality risk. Third, we fit linear regression models for the viral load as a continuous variable to estimate the results. Fourth, we reexamined the hazard ratios using a time-dependent Cox proportional risk model.

## Results

### Baseline Characteristics of the Participants

A total of 179 participants (79 immediately switched to the PIs, 35 immediately switched to the NNRTIs, and 65 switched to other NNRTIs and then to PIs) were included in our study. The initial conditions of people living with HIV among the 3 switched ART groups were comparable except for the age, CD4(+) T cell counts, and viral load (Multimedia Appendix 3). Of the 179 participants, 138 (77.1%) were male, 90 (50.3%) were married, and 91 (50.8%) were younger than 40 years at baseline (Table 1). Heterosexual transmission was the dominant transmission route (72.6%). About 24.6% of the participants achieved higher than a senior high school level education. Of the participants, 19 (10.6%) had other sexually transmitted diseases (STDs), 18 (6.9%) had tuberculosis, and 110 (61.5%) had CRF_01AE HIV-1 subtype (Table 1). Additionally, 145 participants (81%) had CD4(+) T cell counts<200, and 114 (63.7%) had viral load ≥10,000 copies/ml (Table 1).

**Table 1:** Baseline characteristics of the study participants.

Baseline characteristics		Overall mean (SD)	Median (IQR) follow-up, month	Mortality (per 1000 person-months）
Overall		179 (100)	90 (80-101.5)	1.73
**Age (years)**				
	≤40	91 (50.8)	93 (85.5-103)	1.22
	>40	88 (49.2)	87.5 (74.75-99.5)	2.30
**Gender**				
	Male	138 (77.1)	92 (80.25-102)	1.66
	Female	41 (22.9)	88 (76-99)	1.95
**Education level**				
	No formal education	9 (5.0)	93 (87-105)	2.39
	Primary or junior high school	126 (70.4)	91 (77.75-102.75)	1.92
	Senior high school or more	44 (24.6)	90 (81.75-98)	1.04
**Marital status**				
	Married	90 (50.3)	89 (79-95)	1.75
	Unmarried/widowed/divorced/separated	89 (49.7)	93 (81.75-104.75)	1.71
**Pattern of transmission**				
	Heterosexual	130 (72.6)	90.5 (80-102.75)	1.76
	Homosexual	36 (20.1)	90 (81.75-98)	1.29
	Other	13 (7.3)	89 (80-98)	2.67
**Had other STDs^a^**				
	Yes	19 (10.6)	92 (82.5-94)	2.56
	No	119 (66.5)	90 (80-101)	1.93
	Unknown	41 (22.9)	91 (76-103)	0.81
**Had been treated for tuberculosis**				
	Yes	18 (10.1)	92.5 (86.25-103)	0
	No	161 (89.9)	90 (79-101)	1.94
**Baseline CD4(+) T (cells/** **μ** **L)**				
	<200	145 (81.0)	90 (77-101)	1.92
	≥200	34 (19.0)	92 (82.25-104.5)	0.96
**Baseline viral load copies/Ml**				
	<10000	65 (36.3)	93 (87-103)	1.53
	≥10000	114 (63.7)	88.5 (77.5-98.75)	1.85
**HIV subtype**				
	CRF01_AE	110 (61.5)	90.5 (80.25-101.75)	1.56
	CRF07_BC	50 (27.9)	89 (75.25-94)	2.37
	Others	19 (10.6)	98.0 (84.5-104.5)	1.15

^a^STD: sexually transmitted disease.

### Follow-up and Mortality

The follow-up time of 179 patients ranged from 30 to 119 months, and the median follow-up time was 90 months. During a follow-up of 15,606 person-months, 27 individuals from the cohort died. The overall mortality was 1.73 per 1000 person-months (Table 1). The observed mortality rates were higher in individuals with lower CD4(+) T cell count and older age at baseline (Table 1).

### Estimated Risk of Mortality

**Figure 1:**
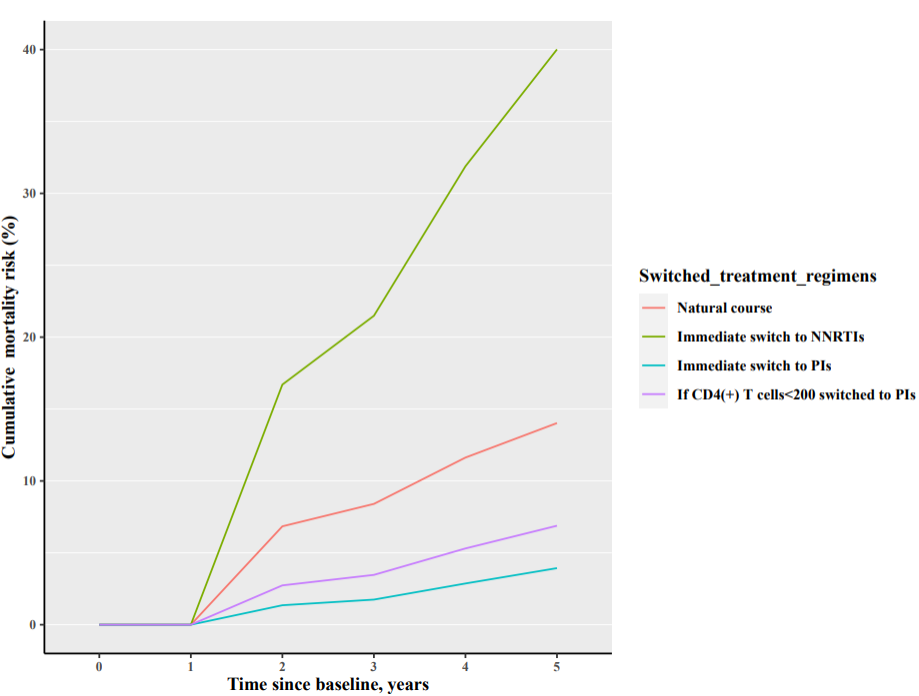
Mean of the mortality outcome for individuals who were tested for HIV-1 genotype resistance from antiretroviral therapy (ART) in Sichuan, China, simulated via the parametric g-formula. NNRTIs: nonnucleoside reverse transcriptase inhibitor–based antiretroviral therapy; PIs: protease inhibitor–based ART.

The estimated 5-year risk of mortality under natural course was 11.62% (95% CI 7.82-17.11). The estimated 5-year risk of mortality of the 3 ART regimen switch scenarios of an immediate switch to NNRTIs, immediate switch to PIs, and switch to PIs if CD4(+) T cells <200 was 31.88% (95% CI 20.79-44.94), 2.87% (95% CI 0.32-7.07), and 5.30% (95% CI 2.07-10.21), respectively (Table 2). The mortality risk for the 4 treatment regimens scenarios increased over time, with the fastest mortality rate for immediate switch to NNRTIs scenario and the slowest rate for immediate switch to PIs scenario (Figure 1).

**Figure 2:**
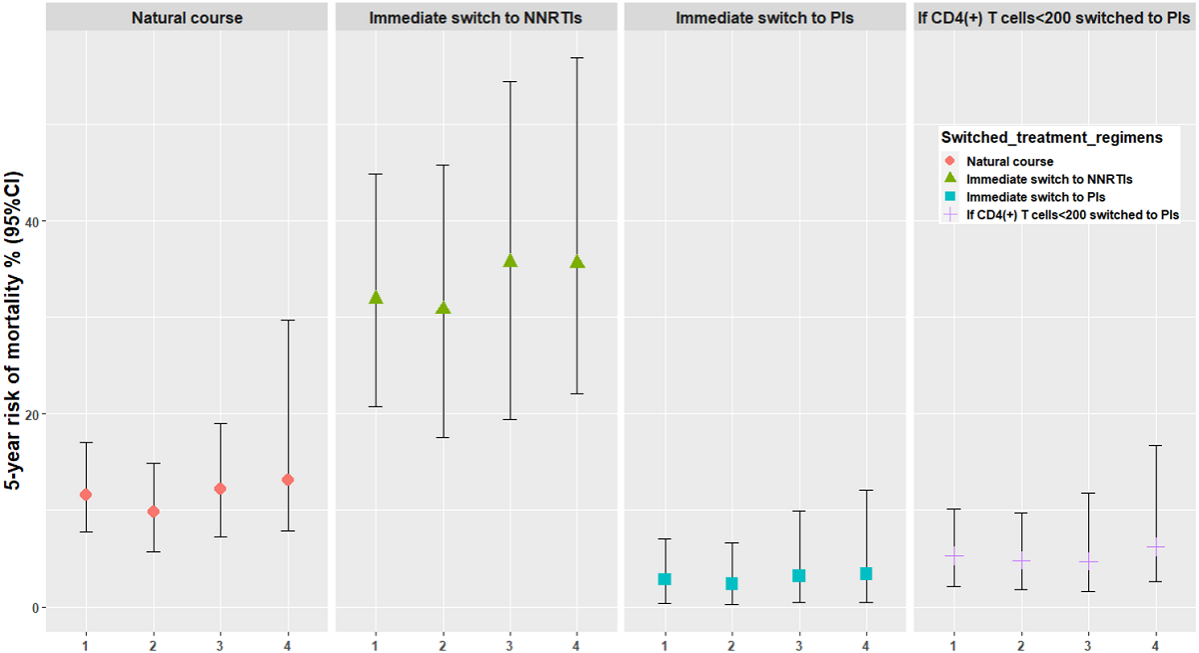
Five-year risk of mortality under 4 analyses. 1: primary analyses (viral load as a binary variable); 2: individuals lower than 60 years at baseline; 3: individuals with a CRF01_AE subtype; 4: viral load as a continuous variable. NNRTIs: nonnucleoside reverse transcriptase inhibitor–based antiretroviral therapy (ART); PIs: protease inhibitor–based ART.

**Figure 3:**
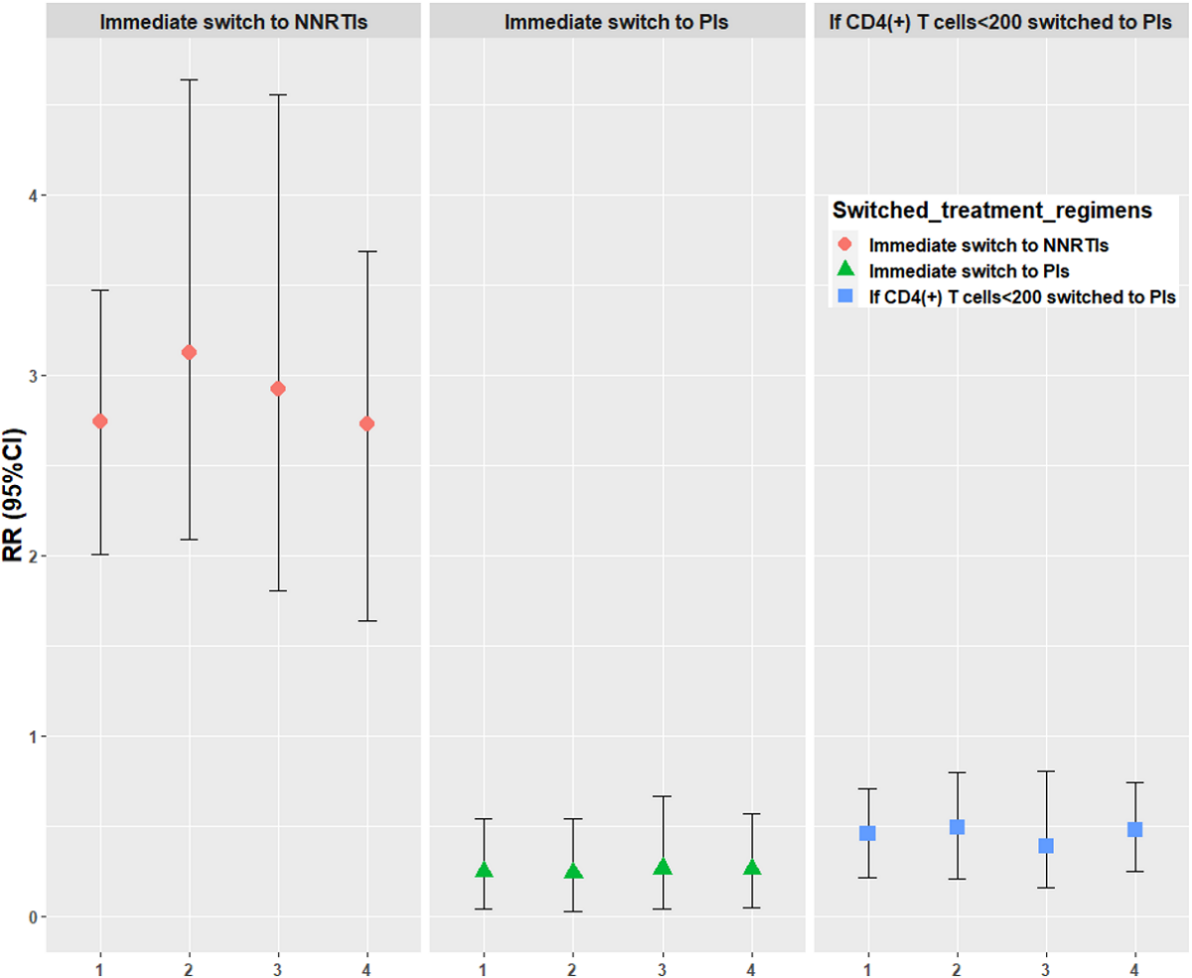
Risk ratio of mortality under 4 analyses. 1: primary analyses (viral load as a binary variable); 2: individuals lower than 60 years old at baseline; 3: individuals with a CRF01_AE subtype; 4: viral load as a continuous variable. NNRTIs: nonnucleoside reverse transcriptase inhibitor–based antiretroviral therapy (ART); PIs: protease inhibitor–based ART; RR: risk ratio.

**Figure 4:**
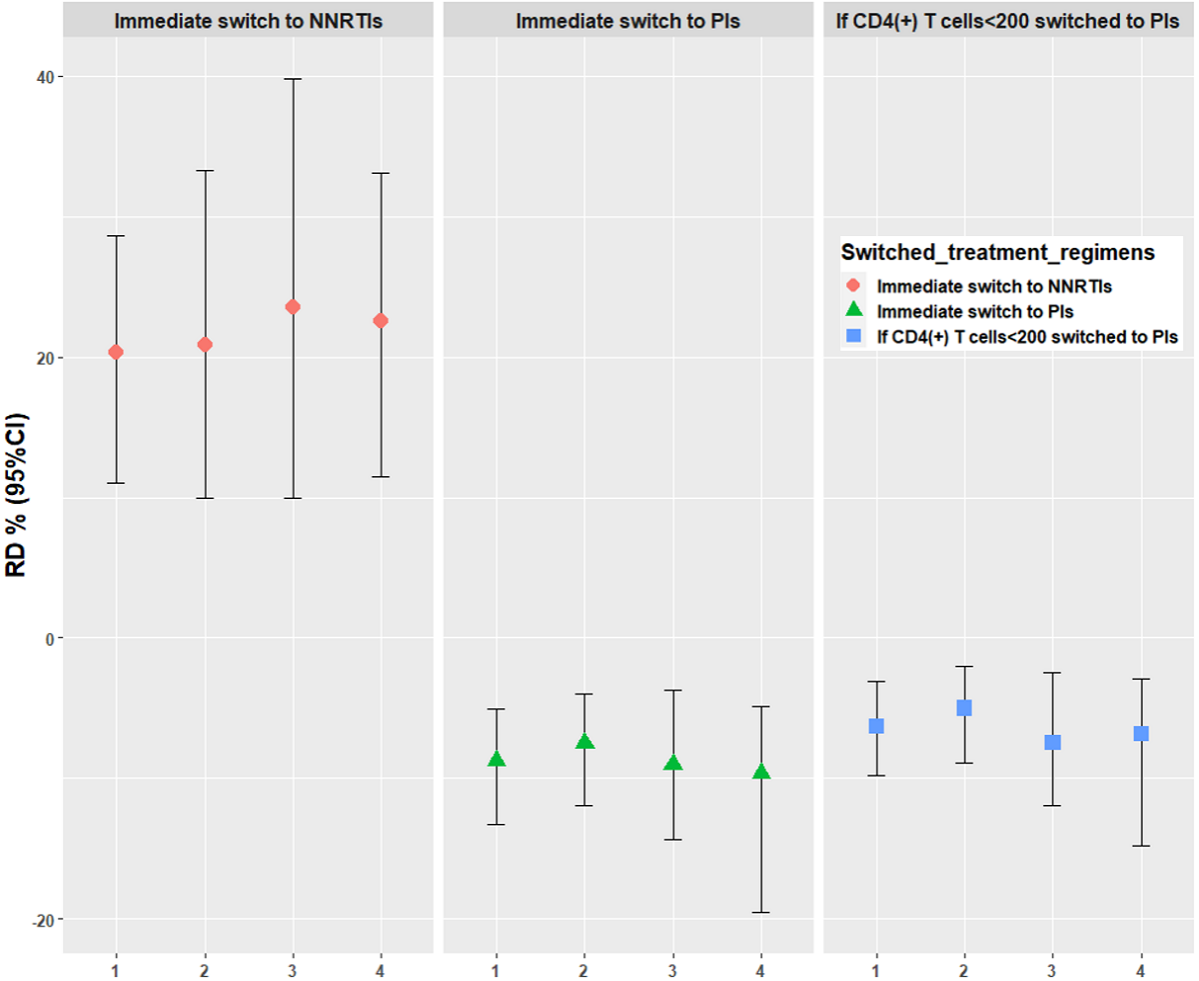
Risk difference of mortality under 4 analyses. 1: primary analyses (viral load as a binary variable), 2: individuals lower than 60 years old at baseline; 3: individuals with a CRF01_AE subtype; 4: viral load as a continuous variable. NNRTIs: nonnucleoside reverse transcriptase inhibitor–based antiretroviral therapy (ART); PIs: protease inhibitor–based ART; RD: risk difference.

Using the parametric g-formula, the RRs of immediate switch to NNRTIs, immediate switch to PIs, and switch to PIs if CD4(+) T cells<200, compared with natural course mortality rate, were 2.74 (95% CI 2.01-3.47), 0.25 (95% CI 0.04-0.54), and 0.46 (95% CI: 0.22-0.71), respectively, and the RDs were 20.26% (95% CI 10.96-28.61), −8.76% (95% CI −13.34 to −5.09), and −6.32% (95% CI −9.75 to −3.11), respectively. The effect of the sensitivity analyses estimates of the 5-year risk of mortality, RRs, and RDs of the 3 ART regimen switch scenarios were robust (Multimedia Appendix 4, Figures 2-4). The Cox proportional risk model results showed the hazard ratio (HR) of mortality among people living with HIV with an immediate switch to PIs (HR=0.11, 95% CI 0.03-0.39), and the switch to other NNRTIs and then to PIs (HR=0.08, 95% CI 0.02-0.33) was lower than those with the immediate switch to NNRTIs (Multimedia Appendix 5).

**Table 2:** Estimated risks of mortality under 4 antiretroviral therapy (ART) switched strategies for individuals tested for HIV-1 genotype resistance from ART in Sichuan from 2011 to 2020^a^.

Switched treatment regimens	5-year risk of mortality (95% CI)	RR^b^, (95% CI)	RD^c^, % (95% CI)
Natural course	11.62 (7.82-17.11)	1 (Ref^d^)	0 (Ref^d^)
Immediate switch to NNRTIs^e^	31.88 (20.79-44.94)	2.74 (2.01-3.47)	20.26 (10.96- 28.61)
Immediate switch to PIs^f^	2.87 (0.32-7.07)	0.25 (0.04-0.54)	−8.76 (−13.34 to −5.09)
If CD4(+) T cells<200 switched to PIs.	5.30 (2.07-10.21)	0.46 (0.22-0.71)	−6.32 (−9.75 to −3.11)

^a^Estimates based on the parametric g-formula adjusted for measured time-varying confounders (CD4(+) T cells count, viral load, and treatment) and baseline characteristics (age, gender, education level, marital status, pattern of transmission, history of sexually transmitted diseases, and history of tuberculosis treatment). Natural course means that the ART regimen is observed without simulated intervention. Natural course mortality was subtracted from estimated mortality for each group.

^b^RR: risk ratio.

^c^RD: risk difference.

^d^Ref: reference object.

^e^NNRTIs: nonnucleoside reverse transcriptase inhibitor–based ART.

^f^PIs: protease inhibitor–based ART.

## Discussion

### Principal Findings

This retrospective cohort study compared the mortality risk of ART switch regimens among people living with HIV with resistance to NNRTIs using the parametric g-formula method. It provided real-world evidence on the efficacy of PIs and NNRTIs switches. Our results indicate that switching to NNRTIs resulted in a higher mortality rate during the 9- year follow-up period. The 2 scenarios showing a switch to PIs were associated with lower mortality rates. This finding suggests that an immediate switch to PIs after confirmation of drug resistance can help reduce the mortality of people living with HIV and HIV-1 drug resistance.

The 5-year risk of mortality was 8.76% lower for an immediate switch to PIs and 6.32% lower when switched to PIs if CD4(+) T cells<200 compared to the natural course group, which means that in a hypothetical cohort of 100 patients with drug resistance, PIs would prevent about 6-8 deaths over 5 years. On the contrary, the 5-year risk of mortality was 20.26% higher for an immediate switch to NNRTIs when comparing the natural course scenario, meaning that NNRTIs would have an increase of 20 deaths over 5 years.

People living with HIV who switched to PIs had a lower mortality risk. Possible reasons for this might be that PIs could cross the resistance gene barrier and act on the resistant strains to inhibit virus replication [35] or that our participants might have had cross-resistance to NNRTIs and NRTI drugs [36]. If only NNRTIs drugs in the original ART regime were replaced (eg, switching NVP to EFV) or the drug type of NRTIs was replaced (eg, switching AZT to TDF), the immunological outcomes did not change due to the cross-resistance. There was a higher mortality risk if participants continued NNRTI-based ART.

A few studies reported that the resistance rates of NNRTIs and NRTIs were 80%-92% and 95%-100% after the failure of the NNRTI-based ART regimen, respectively [37,38]. With the therapy time prolonged, the resistance mutations of the reverse transcriptase inhibitor would accumulate, leading to severer cross-resistance. If the participants still switched to the NNRTIs, cross-resistance of the strains resulted in no significant improvement in follow-up therapy. Although LPV/r in China is free, the first condition for switching to PIs regimen is NNRTI resistance due to the limited resources of LPV/r in most provinces [20,39,40]. Therefore, it is important to monitor and detect drug-resistant patients to NNRTIs in a timely manner and switch to PIs to successfully inhibit the replication of NRTI- and NNRTI-mutant viruses. This monitoring will improve the virological and immunological effects, consequentially reducing mortality. Additionally, switching to PIs if CD4(+) T cells <200 is also effective for reducing the mortality risk if switching immediately without assessing cell count is impossible in some resource-limited areas.

### Strengths and Limitations

Our study has 2 strengths. First, we simulated interventions to evaluate the risk, risk difference, and risk ratio of mortality by contrasting estimates from idealized study settings with those from more realistic settings. Compared with standard statistical calculations, the parametric g-formula can be more easily used to evaluate the causal effect of complex interventions [41]. In particular, dynamic treatments [42] and joint interventions considering multiple factors can be explored naturally with this method. Second, to our knowledge, this is the first study to compare the mortality difference between switching to other NNRTIs and switching to PIs among people living with HIV with NNRTIs resistance, avoiding the ethical issues of randomized clinical trials.

Nonetheless, several limitations should be considered. First, as in all nonrandomized studies, our approach provided consistent (unbiased) estimates of the cumulative incidence of mortality under several assumptions: all variables (eg, CD4(+) T cell count, viral load, treatment, and death) were measured without error; patients' ART treatment at different CD4(+) T cell counts are exchangeable within levels of measured covariates, in that there are no unmeasured confounding variables [30]. However, we did not collect ART adherence information, which may influence the decision to switch ART regimens in patience with resistance to NNRTIs and may have biased our estimates. Second, the parametric g-formula requires that all models be correctly specified. This condition cannot be guaranteed, but it seems plausible because our models resulted in simulated data sets with means of the outcome and time-varying covariates similar to those in the original data (Multimedia Appendix 2). Third, although we collected the data of all patients who received HIV-1 genotype resistance, were confirmed to have HIV-1 drug resistance, and could be followed up, only 179 participants were included in our research. The small sample size may have led to low test efficiency. Moreover, many patients may not have receive HIV-1 genotype resistance between 2011 and 2014 since the drug resistance monitoring was only conducted in about 20% of people living with HIV, which may induce potential selection bias and influence the extrapolation of our results.

### Conclusion

Our study found that the PI regimen helped improve the survival time of people living with HIV and HIV-1 drug resistance. More efforts should be conducted to detect HIV-1 drug resistance earlier to ensure timely regimen switches, thereby maximizing the benefit of early switch ART regimens for people living with HIV and HIV-1 drug resistance and reducing their mortality.

## Appendix

Multimedia Appendix 1 Directed acyclic graph.

Multimedia Appendix 2 Mean of the mortality outcome and time-varying variables in persons living with HIV and HIV-1 drug resistance.

Multimedia Appendix 3 Basic characteristics of the population with 2 antiretroviral therapy (ART) regimens at the observed starting point.

Multimedia Appendix 4 Estimated risks of mortality under 4 antiretroviral therapy (ART) switched strategies for 3 sensitivity analyses.

Multimedia Appendix 5 Hazard ratios (HRs) and 95% CIs for association between mortality and antiretroviral therapy (ART) switched strategies according to Cox proportional hazards model.

## Abbreviations

ART: antiretroviral therapy
AZT: zidovudine
EFV: efavirenz
FPV/r: fosamprenavir/ritonavir
HIVDR: HIV drug resistance
HR: hazard ratio
ISLE: International Institute of Spatial Lifecourse Epidemiology
LPV/r: lopinavir/ritonavir
NNRTI: nonnucleoside reverse transcriptase inhibitor
NRTI: nucleoside reverse transcriptase inhibitor
NVP: nevirapine
NFV: nelfinavir
PI: protease inhibitor
RR: risk ratio
RD: risk difference
TDF: tenofovir
TPV/r: tipranavir/ritonavir
3TC: lamivudine
