# Supplementary material for: Effect of Switching Antiretroviral Treatment Regimen in Patients With Drug-Resistant HIV-1 Infection: Retrospective Observational Cohort Study
Source: JMIR Public Health Surveill. 2022 Jun 24;8(6):e33429. doi: 10.2196/33429 (PMC9270715; doi:10.2196/33429)
Supplement: Multimedia Appendix 1 [file publichealth_v8i6e33429_app1.docx]

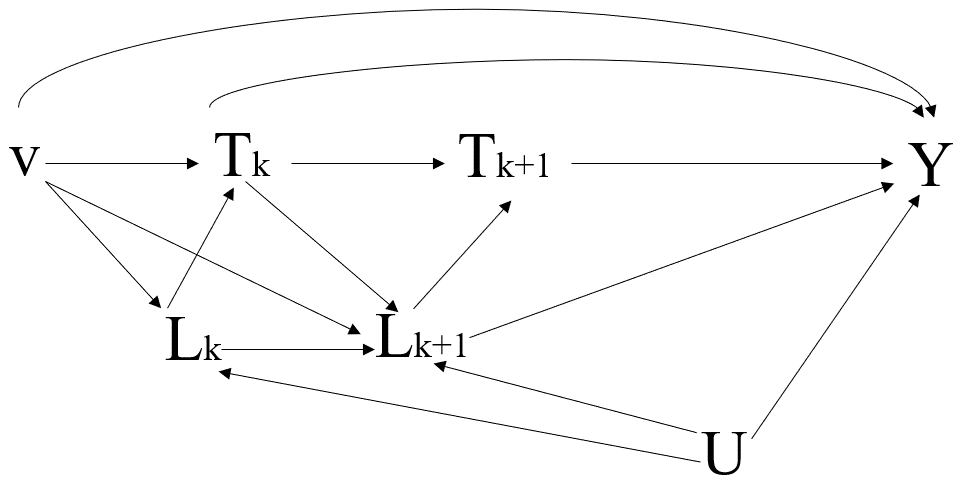


Multimedia Appendix 1. Directed acyclic graph depicting the assumed relationships between baseline covariates V (i.e., age, gender, and education level ), time-varying treatment (ART Regimens ), time-varying confounder L(i.e., CD4, VL), and outcome Y (death). Unmeasured covariates are represented by U, while subscripts (k) denote different time points for time- varying covariates.
