# Supplementary material for: Effect of Switching Antiretroviral Treatment Regimen in Patients With Drug-Resistant HIV-1 Infection: Retrospective Observational Cohort Study
Source: JMIR Public Health Surveill. 2022 Jun 24;8(6):e33429. doi: 10.2196/33429 (PMC9270715; doi:10.2196/33429)
Supplement: Multimedia Appendix 2 [file publichealth_v8i6e33429_app2.docx]

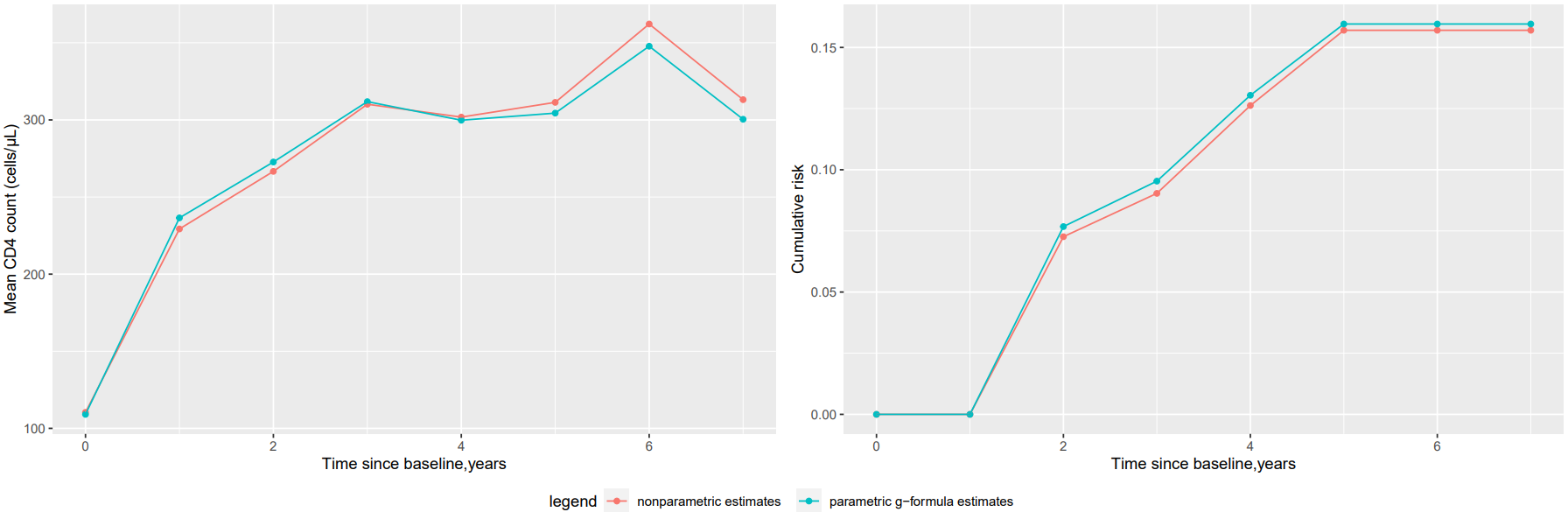


Multimedia Appendix 2. Mean of the mortality outcome and time-varying variables in PLWH with HIV-1 drug resistance: observed (red line) and simulated via the parametric g-formula (green line).
