# Supplementary material for: Effect of Switching Antiretroviral Treatment Regimen in Patients With Drug-Resistant HIV-1 Infection: Retrospective Observational Cohort Study
Source: JMIR Public Health Surveill. 2022 Jun 24;8(6):e33429. doi: 10.2196/33429 (PMC9270715; doi:10.2196/33429)
Supplement: Multimedia Appendix 3 [file publichealth_v8i6e33429_app3.docx]

Multimedia Appendix 3. The basic characteristics of the population with two ART regimens at the observed starting point.

| *Baseline Characteristics* | Immediate switch to PIs, (n=79) | Immediate switch to NNTIs, (n=35) | Switch to other NNRTIs and then to PIs , (n=65) | P-value |
| --- | --- | --- | --- | --- |
| Age (year) |  |  |  |  |
| ≤40 | 46 (58.23) | 12 (34.29) | 33 (50.77) | 0.031 |
| ＞40 | 33 (41.77) | 23 (65.71) | 32 (49.23) |  |
| Gender |  |  |  |  |
| Male | 57 (72.15) | 28 (80.00) | 53 (81.54) | 0.513 |
| Female | 22 (27.85) | 7 (20.00) | 12 (18.46) |  |
| Education Level |  |  |  |  |
| Illiteracy | 4 (5.06) | 0 (0.00) | 5 (7.69) | 0.056 |
| Primary or junior high school | 51 (64.56) | 30 (85.71) | 45 (69.23) |  |
| Senior high school or more | 24 (30.38) | 5 (14.29) | 15 (23.08) |  |
| Marital status |  |  |  |  |
| Married | 39 (49.37) | 20 (57.14) | 30 (46.15) | 0.573 |
| Unmarried /Widowed/Divorced/Separated | 40 (50.63) | 15 (42.86) | 35 (53.85) |  |
| Patterns of transmission |  |  |  |  |
| Heterosexuality | 54 (68.35) | 28 (80.00) | 48 (73.85) | 0.443 |
| Homosexuality | 18 (22.78) | 5 (14.29) | 13 (20.00) |  |
| Others | 7 (8.87) | 2 (5.71) | 4 (6.15) |  |
| Had other STDs |  |  |  |  |
| Yes | 10 (12.66) | 3 (8.57) | 6 (9.23) | 0.314 |
| No | 47 (59.49) | 26 (74.29) | 46 (70.77) |  |
| Unknown | 22 (27.85) | 6 (17.14) | 13 (20.00) |  |
| Had been treated for tuberculosis |  |  |  |  |
| Yes | 7 (8.86) | 2 (5.71) | 9 (13.85) | 0.843 |
| No | 72 (91.14) | 33 (94.29) | 56 (86.15) |  |
| Baseline CD4 (+) T (cells/*μ*L) |  |  |  |  |
| <200 | 28 (35.44) | 24 (68.57) | 36 (55.38) | 0.002 |
| ≥200 | 51 (64.56) | 11 (31.43) | 29 (44.62) |  |
| Baseline viral load  copies/mL |  |  |  |  |
| <10000 | 74 (93.67) | 19 (54.29) | 59 (90.77) | <0.001 |
| ≥10000 | 5 (6.33) | 16 (45.71) | 6 (9.23) |  |
| HIV subtype |  |  |  |  |
| CRF01_AE | 46 (58.23) | 17 (48.57) | 47 (72.31) | 0.233 |
| CRF07_BC | 24 (30.38) | 13 (37.14) | 13 (20.00) |  |
| Others | 9 (11.39) | 5 (14.29) | 5 (7.69) |  |

STDs: sexually transmitted diseases
