# Supplementary material for: Effect of Switching Antiretroviral Treatment Regimen in Patients With Drug-Resistant HIV-1 Infection: Retrospective Observational Cohort Study
Source: JMIR Public Health Surveill. 2022 Jun 24;8(6):e33429. doi: 10.2196/33429 (PMC9270715; doi:10.2196/33429)
Supplement: Multimedia Appendix 4 [file publichealth_v8i6e33429_app4.docx]

Multimedia Appendix 4. Estimated risks of mortality under four ART switched strategies for 3 sensitivity analyses.

| **Analyses** | 5-year risk of mortality % (95% CI) | RR (95% CI) | RD, % (95% CI) |
| --- | --- | --- | --- |
| **Individuals lower than 60 years old at baseline*** |  |  |  |
| Natural course | 9.85 (5.72, 14.93) | 1(Ref.) | 0(Ref.) |
| Immediate switch to NNRTIs | 30.76 (17.62, 45.84) | 3.12 (2.09, 4.64) | 20.91 (10.01, 33.31) |
| Immediate switch to PIs | 2.36 (0.21, 6.60) | 0.24 (0.03, 0.54) | -7.50 (-11.95, -4.00) |
| If CD4(+) T cells<200 switched to PIs | 4.84 (1.77, 9.73) | 0.49 (0.21, 0.80) | -5.01 (-8.87, -1.97) |
| **Individuals with a CRF01_AE subtype*** |  |  |  |
| Natural course | 12.20 (7.24, 18.98) | 1(Ref.) | 0(Ref.) |
| Immediate switch to NNRTIs | 35.68 (19.41, 54.46) | 2.92 (1.80, 4.55) | 23.47 (10.00, 39.75) |
| Immediate switch to PIs | 3.21 (0.43, 9.96) | 0.26 (0.04, 0.66) | -9.00 (-14.27, -3.69) |
| If CD4(+) T cells<200 switched to PIs | 4.72 (1.58, 11.78) | 0.39 (0.16, 0.81) | -7.48 (-11.92, -2.48) |
| **viral load as a continuous variable*** |  |  |  |
| Natural course | 13.05 (7.89, 29.71) | 1(Ref.) | 0(Ref.) |
| Immediate switch to NNRTIs | 35.64 (22.11, 56.91) | 2.73 (1.64, 3.68) | 22.59 (11.45, 33.13) |
| Immediate switch to PIs | 3.39 (0.50, 12.13) | 0.26 (0.04, 0.57) | -9.66 (-19.46, -4.89) |
| If CD4(+) T cells<200 switched to PIs | 6.23 (2.68, 16.69) | 0.48 (0.25, 0.74) | -6.82 (-14.83, -2.88) |

Note: natural course means that the ART regimen is observed without simulated intervention. ART: antiretroviral therapy; PIs: protease inhibitors based ART; NNRTIs: non-nucleoside reverse transcriptase inhibitors based ART; RR: Risk Ratio; RD: Risk Difference (RD: no interference mortality was subtracted from estimated mortality for each group)

*Estimates based on the parametric g-formula adjusted for measured time-varying confounders (CD4 count, viral load and treatment) and baseline characteristics (age, gender, education level, marital status, patterns of transmission, the history of sexually transmitted diseases, and the history of tuberculosis treatment).
