# Supplementary material for: Effect of Switching Antiretroviral Treatment Regimen in Patients With Drug-Resistant HIV-1 Infection: Retrospective Observational Cohort Study
Source: JMIR Public Health Surveill. 2022 Jun 24;8(6):e33429. doi: 10.2196/33429 (PMC9270715; doi:10.2196/33429)
Supplement: Multimedia Appendix 5 [file publichealth_v8i6e33429_app5.docx]

Multimedia Appendix 5. HRs and 95% CIs for association between mortality and ART switched strategies according to Cox proportional hazards model.

| ART Regimens | β | SE | Wald | HR (95% CI) | *P*值 |
| --- | --- | --- | --- | --- | --- |
| Immediate switch to NNRTIs |  |  |  | 1(Ref.) |  |
| Immediate switch to PIs | -2.24 | 0.63 | -3.40 | 0.11 (0.03, 0.39) | <0.001 |
| Switch to other NNRTIs and then to PIs | -2.54 | 0.66 | -3.47 | 0.08 (0.02, 0.33) | <0.001 |

ART: antiretroviral therapy; PIs: protease inhibitors based ART; NNRTIs: non-nucleoside reverse transcriptase inhibitors based ART; Estimates based on the Cox proportional hazards model adjusted for CD4 (+) T cells count, viral load, age, gender, education level, marital status, patterns of transmission, the history of sexually transmitted diseases, and the history of tuberculosis treatment.
